# The Matron's Department

**Published:** 1905-10-28

**Authors:** 


### THE MATRON'S DEPARTMENT.

### XI.—BEDDING AND LINEN.

No branch of hospital work demands more careful attention to detail than this. But to the really domestic mind the linen cupboard is a source of continual gratification, and the duties which it entails are a pleasant refuge from harassing cares.

It is among the early duties of the matron on her appointment to make herself acquainted with the condition of the bedding and house linen, and check it through with the inventory. Should there be no inventory she should have one compiled without delay, and should set herself systematically and by degrees to make good deficiencies. It is a great mistake for a new matron to start with a sense of contempt for the makeshifts of her predecessor. She is not compelled to adopt them, but she will often herself be reduced to make contrivances, and may come in time to realise that it is quite easy to do without much which she has learnt to consider absolutely indispensable. But with tact and in time she ought to succeed in bringing this whole department to a high standard of efficiency, and in demonstrating that the truest economy lies in having enough and to spare.

Hair-mattresses, five or six inches thick, on straw palliasses are usually provided for the patients' use and it will be found an economy should space permit, to have these re-made on the premises. The covers need washing not only after bad cases but periodically, and the hair must be disinfected and teased before the mattresses are remade. The hair bolsters, and feather or flock-pillows, too, need constant attention, and it is more satisfactory, as well as more economical, to employ an upholstress to work by the day than to send these articles out to be repaired. A supply of extra pillows, bolsters, and mattresses must be available for each ward and ready of access. The following average supply of linen should be found sufficient for all purposes, though in surgical and in children's wards a further supply of draw-sheets may be necessary.

| *Per Bed.* | *Per Ward* (24 to 30 beds). |
|---|---|
| 4 Sheets. | 24 Doctors' towels. |
| 3 Draw-sheets. | 12 Basin cloths. |
| 3 Blankets. | 12 Bedpan cloths. |
| 1 Counterpane or quilt. | 12 Teacloths. |
| 3 Pillow-cases. | 12 Roller-towels. |
| | 12 Dusters. |
| | 4 Tablecloths. |

The sheets are best made of good unbleached linen sheeting, and with proper treatment they should last six years before being cut up to recommence their career as draw-sheets. They should be stamped at the top and bottom and in the middle with the date and number of the ward. The sister of each ward is responsible for the order in which her linen cupboard is kept and should herself keep the key and give out what is wanted. When these cupboards are not kept locked there is continual coming and going to get necessary articles and the sister can never be sure what she has in stock. Constant supervision on the part of the matron is required to ensure that the linen is fairly used and always in good condition. In purchasing cloths for the ward there should be care to choose a conspicuous difference in texture among those required for distinct purposes, so that they are never confused one with another. At least twice a year there must be a stock-taking or review of the linen in each ward, when the list is gone through and checked with the sister. One of these occasions will be at the time of the annual cleaning, when the ward is closed, and every deficiency should then be made good, worn articles removed, and new ones supplied. Old quilts and blankets make excellent material for use instead of house-flannel, and there are many ways in which the old linen may be turned to account. Most of the bandages used in Paris hospitals are prepared by old women from the almshouses out of the disused bed-linen and other articles in these establishments, and when all has been turned to account a sum of from 40,000 frs. to 50,000 frs. is made every year by selling the rags which are left over. It is certain that much more could be done in English hospitals to turn waste materials to good account than is often attempted.

A separate linen-cupboard must be assigned to the nurses' home or nurses' quarters. The usual allowance to each bed is four sheets, four blankets, two pillow-cases, four towels, with counterpane and toilet cover. The allowance for servants' rooms is the same.

In addition to the linen used in the nurses' quarters, an amount varying according to the size of the institution will be required for general use. It is these articles often indiscriminately used in the kitchen and offices which are most liable to get lost or misused, and it is very necessary that their destination should be clearly defined, and that they should not only be given out but given in under system. The matron or her delegate should give out on Saturday morning the linen for the week from a list of requisitions, and should hold the head housemaid responsible for checking through with this list all the articles given out before they are sent to the wash. Kitchen cloths and such articles as towels for out-patients and lavatories should be given out and collected daily. Two tablecloths a week should be allowed to each table, including the kitchen, and care should be taken that they are neatly folded in the same creases and kept distinct. It avoids confusion to keep them, when not in use, in the table drawers to which they belong, and they should never on any account be allowed to remain on the table all night. When the carving is done at table a napkin should be placed under the dish and an extra napkin should be available in the event of stains appearing on a clean cloth. These trifles, too often neglected, are of considerable importance in keeping up a bright appearance at meals, and the housemaid's pride in a well-kept table is a very different thing to the weary contempt with which a soiled and crumpled cloth will be flung on crooked. It is certain that clean scrubbed deal would make a more appetising background to a meal than some neglected and untidy table garniture in institutions. When napkins are provided—a point which we venture to think is of small importance compared with general smartness—they should be kept in numbered rings, and, if possible, in the table-drawers.

The making and mending of linen is a process never ending. In large institutions where there is a properly-fitted linen-room with a linen-room maid attached, it is comparatively simple. But this is the exception rather than the rule, and the matron may often be compelled to depend on odds-and-ends of time contributed by persons with other duties. She must appoint a competent person to give out all articles to be mended or made, with the necessary supplies of cotton, tape, buttons, etc., and examine every completed article before it is passed into use. When there is more to be done than the staff can cope with, the matron will find plain needlework eagerly undertaken by outside workers in connection with some of the parochial organisations. Under proper regulations this kind of work can be done equally well by the piece, and when the cost of boarding and housing, and the narrow limits of space in the institution are taken into consideration, this arrangement will appear to combine both economy and convenience.

The outlay on bedding and linen must necessarily vary from year to year. A linen book should be kept in which all additions are entered, with a memorandum of what is from time to time discarded, and this should be available for reference as to the expenditure of the year.

